# E-cigarette attitudes and behaviours amongst 15-30-year-olds in the UK

**DOI:** 10.1093/pubmed/fdad138

**Published:** 2023-07-31

**Authors:** Ana-Catarina Pinho-Gomes, Joseph A Santos, Alexandra Jones, Sudhir Raj Thout, Simone Pettigrew

**Affiliations:** The George Institute for Global Health, School of Public Health, Imperial College London, 58 Wood Lane London, W12 7RZ, UK; Institute of Health Informatics, University College London, 222 Euston Road London, NW1 2DA, UK; The George Institute for Global Health, University of New South Wales, Level 5, 1 King Street Newtown 2042, Sydney, NSW, Australia; The George Institute for Global Health, University of New South Wales, Level 5, 1 King Street Newtown 2042, Sydney, NSW, Australia; The George Institute for Global Health, 308, Third Floor, Elegance Tower, Plot No. 8, Jasola District Centre New Delhi 110025, India; The George Institute for Global Health, University of New South Wales, Level 5, 1 King Street Newtown 2042, Sydney, NSW, Australia

**Keywords:** e-cigarettes, smoking, survey, young people

## Abstract

**Background:**

The use of e-cigarettes has been rising in the UK, particularly by young people. This study investigated behaviours, attitudes and beliefs about e-cigarettes amongst 15–30-year-olds in the UK.

**Methods:**

An online survey was administered to a sample of 1009 15–30-year-olds in the UK.

**Results:**

About one in five participants currently used e-cigarettes at least monthly, with 1 in 10 using them daily. Amongst those using e-cigarettes at least monthly, 90% had used e-cigarettes containing nicotine. E-cigarettes were mainly obtained from vape shops and used at home. Having friends who used e-cigarettes and using them to help quit/reduce smoking were the most common reasons for vaping. About half of participants had been exposed to e-cigarette advertising, especially online, and warning labels on e-cigarettes. Most participants agreed that e-cigarettes are addictive (75%), help people quitting smoking (64%) and are bad for health (63%). Previous or current tobacco smokers were 9 and 22 times more likely to use e-cigarettes than never smokers, respectively. Perceiving e-cigarettes as harmful was associated with a 40% lower likelihood of use.

**Conclusion:**

Raising awareness on the uncertain long-term consequences of vaping and regulation of marketing and sales are crucial to protect young people in the UK.

## Introduction

The prevalence of tobacco smoking has been steadily declining over the past decades in the UK.^1^ In 2021, 6.6 million people or 13.3% of people aged 18 years and over smoked cigarettes, which is the lowest proportion of current smokers since 2011.^1^ This reduction resulted from comprehensive public health policies and programmes, such as restrictions on marketing and selling of tobacco products and their use in public environments, together with a reduction in social acceptability of smoking motivated by growing concerns about its detrimental effect on health.^2^ The impact of public health policies has been increased by the development and implementation of diverse pharmacological therapies to support smoking cessation, including a variety of nicotine replacement products.^3^

Electronic cigarettes, also known as e-cigarettes, are battery-operated devices invented in 2003 as an alternative to tobacco smoking.^4^ In addition to the battery component, an e-cigarette comprises an atomiser and a cartridge containing either a nicotine or a non-nicotine solution. When the device is operated, the battery heats the liquid in the cartridge and the atomiser vaporizes the liquid and emits a vapour (hence, the common designation of ‘vaping’) that the user inhales. E-cigarettes have been promoted as an effective aid to quit or reduce tobacco smoking because they may be less harmful than tobacco products, at least in the short term.^5^ However, whilst the updated tobacco guidelines issued by the National Institute for Health and Care Excellence (NICE) in the UK recommend the use of e-cigarettes to support smoking cessation in adults, they acknowledge the uncertainty on the short- and long-term health effects of e-cigarettes, especially for children, young people and pregnant women.^6^

The efficacy of e-cigarettes compared with other forms of nicotine replacement therapy in promoting and sustaining smoking cessation and treating dependence is controversial internationally.^7^ First, the chemicals contained in the solution and vapour may have harmful effects on the lungs, such as vaping-associated lung injury.^8^ Second, most e-cigarettes contain nicotine and hence are at least as addictive as regular cigarettes.^9^ Marketing of e-cigarettes as safer alternatives to tobacco together with their additive potential could promote their use and facilitate the transition to more harmful forms of smoking, such as tobacco.^10^ These concerns underpin the regulation of the composition, marketing and sales of e-cigarettes. For instance, in the UK, chemicals that have been associated with vaping-associated lung injury are not allowed in e-cigarette liquids,^8^^,^^11^ and marketing and advertising of e-cigarettes, particularly to young people, is prohibited.^12^

Notwithstanding public health information campaigns promoting e-cigarettes solely as a smoking cessation aid rather than as a harmless smoking alternative,^13^ the popularity of e-cigarettes has been steadily rising for the past years in the UK.^1^ In 2021, it was estimated that 4 million or 7.7% of people aged 16 years and over currently used an e-cigarette daily or occasionally, which represents an increase on the estimate of 6.4% in 2020.^1^ Furthermore, 2021 was the first year since data collection began in 2014 that young people aged 16–24 years reported the highest proportion of vapers (daily and occasional) at 11.1% in comparison to other age groups.^1^ Amid growing concerns about the impact of e-cigarettes, understanding what is driving the upward trend of their use by young people is crucial to inform public health policy and interventions. Therefore, this study aimed to explore a wide range of vaping-related exposures, attitudes and behaviours amongst 15–30-year-olds in the UK.

## Methods

### Participants

As part of a larger international study,^14^ an ISO-accredited social research agency (Pureprofile) was commissioned to recruit a national sample of ~1000 residents in the UK aged 15–30 years from their web panel. Quotas were applied to ensure the sample had a proportionate distribution between genders and year groups. This quota system was designed to obtain a broadly representative sample and achieved adequate power to detect differences between demographic groups. Between October and November 2021, potential respondents were sent an e-mail invitation to complete an online survey taking ~20 min. Participants were explained the topic and purpose of the study by a participant information letter once they clicked on the link to access the survey. The study was approved by a university Human Research Ethics Committee and all respondents provided informed consent.

### Survey

The survey included questions related to respondent demographics (gender, age, income, education and postcode to assess geographical distribution), vaping/smoking products used,^15–17^ how e-cigarettes are obtained^18^ and where they are used (new question developed for this survey), motivations for vaping,^17^^,^^19^ exposure to others’ vaping behaviours,^17^^,^^20^ exposure to e-cigarette advertising^17^ and beliefs about e-cigarettes.^21^^,^^22^ The questions were based on the referenced sources and adapted as required considering the purpose and target population of the survey. To equivalize item wording across substances, the e-cigarette and tobacco use items asked respondents to report if they ‘Never used’, ‘Previously used’ or ‘Currently use’ a range of products, including e-cigarettes, e-hookahs/pipes, cannabis e-cigarettes, tobacco cigarettes, cigars and pipes. Use was described in terms of ‘even just once or twice’.

### Data analysis

Descriptive analyses were carried out for the included variables and stratified by gender, age and smoking status. Differences between subgroups were tested using chi-squared test. Because of the large number of comparisons, a *P*-value < 0.001 was considered statistically significant. For the analysis of behaviours, only participants who used e-cigarettes at least once monthly were included. For the analyses of exposures and beliefs, participants who said they did not know about e-cigarettes were excluded. Multivariable logistic regression was used to identify factors associated with ever (i.e. previous or current) e-cigarette use. Variables included in the regression were respondent gender, age, education, income and tobacco use status; perceived harmfulness and addictiveness (continuous variable); numbers of family members and friends who use e-cigarettes; and exposure to e-cigarette advertising (binary variable). The latter was coded as exposure to at least one of the four assessed forms of e-cigarette advertising (television/cinema/streaming services, internet, social media and corner stores/supermarkets/petrol stations). The significance threshold for the regression analysis was *P* < 0.05.

## Results

Overall, 1009 participants were included in this study, of whom 520 (51.5%) were women and 19 (1.9%) were non-binary (Table S1). Students accounted for 252 (25%) of the participants. The highest qualifications achieved by most participants were primary or secondary school (486, 48.2%) and university (339, 33.6%). About a third of the participants were from low- and medium-income groups (328, 32.5% and 303, 30.0%) and about one quarter from high-income groups (236, 23.4%). A total of 278 (27.6%) participants were current e-cigarette users and 154 (15.3%) were dual users of tobacco and e-cigarettes.

### E-cigarette-related behaviours and motivations amongst at least monthly current e-cigarette users

Amongst the 1009 participants, 222 (22.0%) reported currently using e-cigarettes at least monthly (Table 1), with no meaningful differences between women (20.6%) and men (22.6%). People aged 15–19 years were less likely to use e-cigarettes at least monthly than older age groups (15.6% for 15–19-year-olds versus 25.8% for 20–25-year-olds and 24.3% for 26–30-year-olds—results not shown in tables). Current smokers were the most likely to use e-cigarettes (44.9%) followed by previous smokers (28.5%), whereas never smokers used them rarely (2.3%) (results not shown in tables).

**Table 1:** E-cigarette-related behaviours and motivations amongst at least monthly current users by gender, age and smoking status—*n* (%)

	Women *n* = 107	Men *n* = 106	Non-binary *n* = 9	15–19 *n* = 51	20–25 *n* = 88	26–30 *n* = 83	Never smoker *n* = 10	Previous smoker *n* = 83	Current smoker i = 129	Overall *n* = 222
Frequency of using e-cigarettes										
At least monthly (but not weekly)	20 (18.7)	8 (7.5)	0 (0.0)	9 (17.6)	13 (14.8)	6 (7.2)	2 (20.0)	15 (18.1)	11 (8.5)	28 (12.6)
At least weekly (but not daily)	38 (35.5)	48 (45.3)	6 (66.7)	20 (39.2)	40 (45.5)	32 (38.6)	4 (40.0)	30 (36.1)	58 (45.0)	92 (41.4)
Daily	49 (45.8)	50 (47.2)	3 (33.3)	22 (43.1)	35 (39.8)	45 (54.2)	4 (40.0)	38 (45.8)	60 (46.5)	102 (45.9)
Ever vaped nicotine e-cigarettes	97 (90.7)	94 (88.7)	8 (88.9)	48 (94.1)	76 (86.4)	75 (90.4)	8 (80.0)	74 (89.2)	117 (90.7)	199 (89.6)
Ever vaped cannabis	37 (34.6)	31 (29.2)	4 (44.4)	19 (37.3)	34 (38.6)	19 (22.9)	2 (20.0)	25 (30.1)	45 (34.9)	72 (32.4)
Medical prescription for e-cigarettes	12 (11.2)	22 (20.8)	0 (0.0)	4 (7.8)	17 (19.3)	13 (15.7)	5 (50.0)	13 (15.7)	16 (12.4)	34 (15.3)
Where e-cigarettes are obtained										
At a vape shop	75 (70.1)	67 (63.2)	4 (44.4)	27 (52.9)	58 (65.9)	61 (73.5)	8 (80.0)	55 (66.3)	83 (64.3)	146 (65.8)
At a corner shop	42 (39.3)	33 (31.1)	3 (33.3)	27 (52.9)	28 (31.8)	23 (27.7)	3 (30.0)	33 (39.8)	42 (32.6)	78 (35.1)
Friend who is over 18 gives it to me	28 (26.2)	33 (31.1)	1 (11.1)	19 (37.3)	19 (21.6)	24 (28.9)	2 (20.0)	19 (22.9)	41 (31.8)	62 (27.9)
Through the internet	28 (26.2)	23 (21.7)	2 (22.2)	14 (27.5)	18 (20.5)	21 (25.3)	2 (20.0)	21 (25.3)	30 (23.3)	53 (23.9)
My parent(s)/legal guardian(s) give them to me	13 (12.1)	19 (17.9)	3 (33.3)	9 (17.6)	14 (15.9)	12 (14.5)	3 (30.0)	13 (15.7)	19 (14.7)	35 (15.8)
At a petrol station	19 (17.8)	14 (13.2)	1 (11.1)	6 (11.8)	13 (14.8)	15 (18.1)	1 (10.0)	14 (16.9)	19 (14.7)	34 (15.3)
At a kiosk in a shopping centre or mall	16 (15.0)	9 (8.5)	0 (0.0)	7 (13.7)	9 (10.2)	9 (10.8)	1 (10.0)	7 (8.4)	17 (13.2)	25 (11.3)
At a tobacconist/tobacco shop	11 (10.3)	13 (12.3)	0 (0.0)	2 (3.9)	9 (10.2)	13 (15.7)	1 (10.0)	5 (6.0)	18 (14.0)	24 (10.8)
My brother/sister give them to me	7 (6.5)	15 (14.2)	1 (11.1)	3 (5.9)	12 (13.6)	8 (9.6)	2 (20.0)	6 (7.2)	15 (11.6)	23 (10.4)
I get someone to buy it for me	15 (14.0)	7 (6.6)	0 (0.0)	11 (21.6)	6 (6.8)	5 (6.0)	1 (10.0)	10 (12.0)	11 (8.5)	22 (9.9)
At a pharmacy or chemist	9 (8.4)	8 (7.5)	0 (0.0)	2 (3.9)	6 (6.8)	9 (10.8)	1 (10.0)	5 (6.0)	11 (8.5)	17 (7.7)
Friend who is under 18 gives it to me	8 (7.5)	7 (6.6)	0 (0.0)	5 (9.8)	6 (6.8)	4 (4.8)	1 (10.0)	6 (7.2)	8 (6.2)	15 (6.8)
I take them from home without my parent(s)/legal guardian(s) permission	6 (5.6)	7 (6.6)	0 (0.0)	5 (9.8)	6 (6.8)	2 (2.4)	2 (20.0)	2 (2.4)	9 (7.0)	13 (5.9)
Opinion about the cost of e-cigarettes										
E-cigarettes are cheaper than tobacco cigarettes	59 (55.1)	64 (60.4)	3 (33.3)	26 (51.0)	50 (56.8)	50 (60.2)	5 (50.0)	51 (61.4)	70 (54.3)	126 (56.8)
E-cigarettes are more expensive than tobacco cigarettes	20 (18.7)	22 (20.8)	4 (44.4)	10 (19.6)	19 (21.6)	17 (20.5)	1 (10.0)	15 (18.1)	30 (23.3)	46 (20.7)
E-cigarettes cost about the same as tobacco cigarettes	18 (16.8)	18 (17.0)	1 (11.1)	6 (11.8)	18 (20.5)	13 (15.7)	3 (30.0)	12 (14.5)	22 (17.1)	37 (16.7)
I don’t know the cost of e-cigarettes compared with tobacco cigarettes	10 (9.3)	2 (1.9)	1 (11.1)	9 (17.6)	1 (1.1)	3 (3.6)	1 (10.0)	5 (6.0)	7 (5.4)	13 (5.9)
Where cigarettes are used										
At home, both inside and outside	76 (71.0)	55 (51.9)	6 (66.7)	31 (60.8)	52 (59.1)	54 (65.1)	4 (40.0)	54 (65.1)	79 (61.2)	137 (61.7)
At parties	43 (40.2)	28 (26.4)	2 (22.2)	21 (41.2)	29 (33.0)	23 (27.7)	1 (10.0)	28 (33.7)	44 (34.1)	73 (32.9)
At other people’s homes	42 (39.3)	24 (22.6)	2 (22.2)	20 (39.2)	30 (34.1)	18 (21.7)	1 (10.0)	24 (28.9)	43 (33.3)	68 (30.6)
At my workplace	27 (25.2)	38 (35.8)	1 (11.1)	12 (23.5)	34 (38.6)	20 (24.1)	3 (30.0)	22 (26.5)	41 (31.8)	66 (29.7)
At home, but only if I am outside	22 (20.6)	29 (27.4)	2 (22.2)	15 (29.4)	21 (23.9)	17 (20.5)	1 (10.0)	23 (27.7)	29 (22.5)	53 (23.9)
At licensed premises (e.g. pubs, clubs)	29 (27.1)	21 (19.8)	2 (22.2)	13 (25.5)	24 (27.3)	15 (18.1)	0 (0.0)	18 (21.7)	34 (26.4)	52 (23.4)

(*Continued*)

**Table 1:** Continued

	Women *n* = 107	Men *n* = 106	Non-binary *n* = 9	15–19 *n* = 51	20–25 *n* = 88	26–30 *n* = 83	Never smoker *n* = 10	Previous smoker *n* = 83	Current smoker i = 129	Overall *n* = 222
At restaurants/cafes	21 (19.6)	22 (20.8)	0 (0.0)	7 (13.7)	21 (23.9)	15 (18.1)	0 (0.0)	13 (15.7)	30 (23.3)	43 (19.4)
On university or other educational facility grounds (excluding school)	21 (19.6)	18 (17.0)	1 (11.1)	11 (21.6)	19 (21.6)	10 (12.0)	3 (30.0)	13 (15.7)	24 (18.6)	40 (18.0)
On school grounds	19 (17.8)	12 (11.3)	0 (0.0)	11 (21.6)	11 (12.5)	9 (10.8)	1 (10.0)	10 (12.0)	20 (15.5)	31 (14.0)
Reasons why e-cigarettes are used										
A friend used them	55 (51.4)	45 (42.5)	3 (33.3)	29 (56.9)^a^	40 (45.5)	34 (41.0)	6 (60.0)	40 (48.2)	57 (44.2)	103 (46.4)
To help me quit smoking regular cigarettes	48 (44.9)	36 (34.0)	5 (55.6)	20 (39.2)	33 (37.5)	36 (43.4)	3 (30.0)	38 (45.8)	48 (37.2)	89 (40.1)
To try to cut down on the number of cigarettes I smoke/smoked	38 (35.5)	37 (34.9)	6 (66.7)	20 (39.2)	32 (36.4)	29 (34.9)	2 (20.0)	25 (30.1)	54 (41.9)	81 (36.5)
I think they are less harmful than regular cigarettes	41 (38.3)	26 (24.5)	0 (0.0)	13 (25.5)	28 (31.8)	26 (31.3)	3 (30.0)	29 (34.9)	35 (27.1)	67 (30.2)
I think they taste better than regular cigarettes	31 (29.0)	27 (25.5)	5 (55.6)	17 (33.3)	26 (29.5)	20 (24.1)	1 (10.0)	28 (33.7)	34 (26.4)	63 (28.4)
To stop me going back to smoking regular cigarettes	27 (25.2)	31 (29.2)	2 (22.2)	10 (19.6)	26 (29.5)	24 (28.9)	2 (20.0)	26 (31.3)	32 (24.8)	60 (27.0)
They are cheaper than regular cigarettes	32 (29.9)	26 (24.5)	2 (22.2)	14 (27.5)	25 (28.4)	21 (25.3)	1 (10.0)	25 (30.1)	34 (26.4)	60 (27.0)
A family member used them	29 (27.1)	17 (16.0)	3 (33.3)	13 (25.5)	18 (20.5)	18 (21.7)	1 (10.0)	18 (21.7)	30 (23.3)	49 (22.1)
They have appealing flavours	25 (23.4)	21 (19.8)	2 (22.2)	14 (27.5)	19 (21.6)	15 (18.1)	0 (0.0)	20 (24.1)	28 (21.7)	48 (21.6)
They don’t smell (unlike regular cigarettes)	24 (22.4)	20 (18.9)	2 (22.2)	10 (19.6)	18 (20.5)	18 (21.7)	0 (0.0)	20 (24.1)	26 (20.2)	46 (20.7)
You can use them in places where regular cigarettes are banned	25 (23.4)	13 (12.3)	1 (11.1)	8 (15.7)	14 (15.9)	17 (20.5)	0 (0.0)	17 (20.5)	22 (17.1)	39 (17.6)
Out of curiosity	22 (20.6)	14 (13.2)	3 (33.3)	20 (39.2)	9 (10.2)	10 (12.0)	2 (20.0)	16 (19.3)	21 (16.3)	39 (17.6)
For entertainment, e.g. for fun, to relax, boredom	22 (20.6)	11 (10.4)	4 (44.4)	18 (35.3)	12 (13.6)	7 (8.4)	2 (20.0)	14 (16.9)	21 (16.3)	37 (16.7)
They seem more acceptable than regular cigarettes	20 (18.7)	15 (14.2)	1 (11.1)	10 (19.6)	19 (21.6)	7 (8.4)	1 (10.0)	15 (18.1)	20 (15.5)	36 (16.2)
Flavours of e-cigarettes used										
Fruit flavour	82 (76.6)	58 (54.7)	6 (66.7)	44 (86.3)	53 (60.2)	49 (59.0)	6 (60.0)	59 (71.1)	81 (62.8)	146 (65.8)
Ice flavours	56 (52.3)	39 (36.8)	5 (55.6)	29 (56.9)	39 (44.3)	32 (38.6)	4 (40.0)	42 (50.6)	54 (41.9)	100 (45.0)
Mint	45 (42.1)	39 (36.8)	3 (33.3)	23 (45.1)	28 (31.8)	36 (43.4)	0 (0.0)	34 (41.0)	53 (41.1)	87 (39.2)
Candy, chocolate, dessert, sweets	51 (47.7)	32 (30.2)	3 (33.3)	21 (41.2)	37 (42.0)	28 (33.7)	4 (40.0)	35 (42.2)	47 (36.4)	86 (38.7)
Menthol	43 (40.2)	38 (35.8)	4 (44.4)	20 (39.2)	33 (37.5)	32 (38.6)	0 (0.0)	31 (37.3)	54 (41.9)	85 (38.3)
Tobacco flavour	32 (29.9)	42 (39.6)	4 (44.4)	14 (27.5)	31 (35.2)	33 (39.8)	2 (20.0)	19 (22.9)	57 (44.2)	78 (35.1)
Mix of tobacco and menthol	19 (17.8)	24 (22.6)	1 (11.1)	7 (13.7)	16 (18.2)	21 (25.3)	1 (10.0)	12 (14.5)	31 (24.0)	44 (19.8)
A non-alcoholic drink (soda/soft drink, energy drinks or other beverages)	19 (17.8)	14 (13.2)	1 (11.1)	9 (17.6)	17 (19.3)	8 (9.6)	0 (0.0)	17 (20.5)	17 (13.2)	34 (15.3)
Coffee	12 (11.2)	17 (16.0)	2 (22.2)	5 (9.8)	9 (10.2)	17 (20.5)	0 (0.0)	11 (13.3)	20 (15.5)	31 (14.0)
An alcoholic drink (wine, whisky, cognac, margarita or other cocktails)	13 (12.1)	16 (15.1)	1 (11.1)	5 (9.8)	14 (15.9)	11 (13.3)	1 (10.0)	10 (12.0)	19 (14.7)	30 (13.5)
Nicotine concentration of e-liquid used										
>3 mg/ml	64 (73.6)	71 (79.8)	4 (80.0)	30 (76.9)	52 (76.5)	57 (77.0)	7 (87.5)	50 (74.6)	82 (77.4)	139 (76.8)
3 mg/ml	22 (25.3)	17 (19.1)	1 (20.0)	9 (23.1)	15 (22.1)	16 (21.6)	1 (12.5)	16 (23.9)	23 (21.7)	40 (22.1)
<3 mg/ml	1 (1.1)	1 (1.1)	0 (0.0)	0 (0.0)	1 (1.5)	1 (1.4)	0 (0.0)	1 (1.5)	1 (0.9)	2 (1.1)

(*Continued*)

**Table 1:** Continued

	Women *n* = 107	Men *n* = 106	Non-binary *n* = 9	15–19 *n* = 51	20–25 *n* = 88	26–30 *n* = 83	Never smoker *n* = 10	Previous smoker *n* = 83	Current smoker i = 129	Overall *n* = 222
Brands of e-cigarettes tried										
JUUL	49 (45.8)	57 (53.8)	3 (33.3)	30 (58.8)^a^	46 (52.3)	33 (39.8)	4 (40.0)	33 (39.8)	72 (55.8)	109 (49.1)
blu	39 (36.4)	51 (48.1)	3 (33.3)	19 (37.3)	35 (39.8)	39 (47.0)^a^	3 (30.0)	32 (38.6)	58 (45.0)	93 (41.9)
SMOK	51 (47.7)	39 (36.8)	2 (22.2)	28 (54.9)^a^	32 (36.4)	32 (38.6)	3 (30.0)	38 (45.8)^a^	51 (39.5)	92 (41.4)
Geek Vape	50 (46.7)	27 (25.5)	4 (44.4)	28 (54.9)^a^	31 (35.2)	22 (26.5)	5 (50.0)	36 (43.4)	40 (31.0)	81 (36.5)
Puff bar	26 (24.3)	21 (19.8)	1 (11.1)	20 (39.2)^a^	17 (19.3)	11 (13.3)	3 (30.0)	21 (25.3)	24 (18.6)	48 (21.6)
Vuse	13 (12.1)	21 (19.8)	0 (0.0)	3 (5.9)	12 (13.6)	19 (22.9)	1 (10.0)	12 (14.5)	21 (16.3)	34 (15.3)
Eleaf	13 (12.1)	11 (10.4)	1 (11.1)	6 (11.8)	9 (10.2)	10 (12.0)	2 (20.0)	11 (13.3)	12 (9.3)	25 (11.3)
Vaporesso	9 (8.4)	13 (12.3)	0 (0.0)	4 (7.8)	8 (9.1)	10 (12.0)	0 (0.0)	10 (12.0)	12 (9.3)	22 (9.9)
Uwell	3 (2.8)	8 (7.5)	0 (0.0)	1 (2.0)	7 (8.0)	3 (3.6)	0 (0.0)	5 (6.0)	6 (4.7)	11 (5.0)
NJOY	5 (4.7)	4 (3.8)	0 (0.0)	1 (2.0)	3 (3.4)	5 (6.0)	0 (0.0)	3 (3.6)	6 (4.7)	9 (4.1)
Other	20 (18.7)	4 (3.8)	1 (11.1)	10 (19.6)	9 (10.2)	6 (7.2)	1 (10.0)	10 (12.0)	14 (10.9)	25 (11.3)
Devices of e-cigarette used										
An e-cigarette device with a refillable tank	68 (63.6)	62 (58.5)	4 (44.4)	33 (64.7)	53 (60.2)	48 (57.8)	5 (50.0)	52 (62.7)	77 (59.7)	134 (60.4)
A disposable e-cigarette (for single use until empty), such as a Puff Bar	69 (64.5)	57 (53.8)	5 (55.6)	38 (74.5)	54 (61.4)	39 (47.0)	7 (70.0)	49 (59.0)	75 (58.1)	131 (59.0)
JUUL or other vaping device where you click in a pod containing e-liquid	65 (60.7)	53 (50.0)	3 (33.3)	31 (60.8)	47 (53.4)	43 (51.8)	3 (30.0)	39 (47.0)	79 (61.2)	121 (54.5)
An e-cigarette that uses replaceable cartridges that you screw on	41 (38.3)	52 (49.1)	2 (22.2)	23 (45.1)	34 (38.6)	38 (45.8)	5 (50.0)	37 (44.6)	53 (41.1)	95 (42.8)
A mod system (an e-cigarette that can be customized by the user with a combination of batteries or other parts)	26 (24.3)	23 (21.7)	1 (11.1)	12 (23.5)	19 (21.6)	19 (22.9)	2 (20.0)	22 (26.5)	26 (20.2)	50 (22.5)

^a^Significant difference between columns for the same variable (sex, age, smoking) at *P* < 0.001. This refers to differences between that column and all other columns for the same variable.

A total of 102 (45.9%) of those who used e-cigarettes at least monthly reported using them daily. Ever use of e-cigarettes containing nicotine was reported by about 9 in 10 of those who used e-cigarettes at least monthly (199, 89.6%), whereas ever use of cannabis via e-cigarettes was reported by about one in three 72 (32.4%). Prescriptions for e-cigarettes were overall uncommon (34, 15.3%) in this group.

Vape shops were the most common place where e-cigarettes were obtained (146, 65.8%), followed by corner shops (78, 35.1%), friends over 18-year-olds (62, 27.9%) and through the internet (53, 23.9%). Over half of those who used e-cigarettes at least monthly considered that e-cigarettes were cheaper than tobacco cigarettes (126, 56.8%) and about one in five considered them to be more expensive (46, 20.7%).

The most common places where e-cigarettes were used were at home (both inside and outside: 137, 61.7%), at parties (73, 32.9%), at other people’s homes (68, 30.6%) and at the workplace (66, 29.7%).The most common reasons for using e-cigarettes were because a friend used them (103, 46.4%), as an aid to stop or reduce the number of tobacco cigarettes (89, 40.1% and 81, 36.5%, respectively), and because they were perceived as less harmful than tobacco cigarettes (67, 30.2%). Favourite flavours were fruit (146, 65.8%) and ice (100, 45.0%). E-liquids with a nicotine concentration over 3 mg/dL were the most commonly used (139, 76.8%). The most common brand was ‘JUUL’ (109, 49.1%), followed by ‘blu’ (93, 41.9%). E-cigarettes with a refillable tank and disposable models were similarly popular amongst e-cigarette users (60.4 and 59.0%, respectively). Disposable devices were particularly common amongst users under 20-year-olds (74.5%).

### E-cigarettes beliefs and external influences

Most participants agreed or strongly agreed that e-cigarettes are addictive (698, 75.1%), contain chemicals (671, 72.2%), contain nicotine (628, 67.5%), help people quitting smoking (597, 64.2%) and are bad for health (584, 62.8%) (Table 2). About two in five participants thought that e-cigarettes were a tobacco product (402, 43.2%). Women were more likely than men to consider e-cigarettes addictive (80.2 versus 69.1%) and harmful for health (65.6 versus 59.1%).

**Table 2:** E-cigarette-related beliefs by gender, age and smoking status—*n* (%)

	Women (*N* = 479)	Men (*N* = 433)	Non-binary (*N* = 18)	15–19 (*N* = 299)	20–25 (*N* = 314)	26–30 (*N* = 317)	Never smoker (*N* = 371)	Previous smoker (*N* = 282)	Current smoker (*N* = 277)	Overall (*N* = 930)
E-cigarettes are addictive	384 (80.2)	299 (69.1)	15 (83.3)	220 (73.6)	240 (76.4)	238 (75.1)	261 (70.4)	231 (81.9)^a^	206 (74.4)	698 (75.1)
E-cigarettes contain chemicals	361 (75.4)	294 (67.9)	16 (88.9)	226 (75.6)	218 (69.4)	227 (71.6)	260 (70.1)	211 (74.8)	200 (72.2)	671 (72.2)
E-cigarettes contain nicotine	325 (67.8)	287 (66.3)	16 (88.9)	192 (64.2)	223 (71.0)	213 (67.2)	218 (58.8)^a^	202 (71.6)	208 (75.1)	628 (67.5)
E-cigarettes help people quit using cigarettes	321 (67.0)	264 (61.0)	12 (66.7)	188 (62.9)	197 (62.7)	212 (66.9)	206 (55.5)^a^	200 (70.9)	191 (69.0)	597 (64.2)
E-cigarettes are bad for your health	314 (65.6)	256 (59.1)	14 (77.8)	200 (66.9)	183 (58.3)	201 (63.4)	241 (65.0)	170 (60.3)	173 (62.5)	584 (62.8)
E-cigarette devices can explode and cause injury	267 (55.7)	215 (49.7)	9 (50.0)	151 (50.5)	164 (52.2)	176 (55.5)	179 (48.2)	148 (52.5)	164 (59.2)^a^	491 (52.8)
E-cigarettes are a tobacco product	215 (44.9)	178 (41.1)	9 (50.0)	105 (35.1)	148 (47.1)	149 (47.0)	136 (36.7)^a^	122 (43.3)	144 (52.0)	402 (43.2)
E-cigarette vapour is harmless water vapour	99 (20.7)	129 (29.8)	4 (22.2)	48 (16.1)^a^	88 (28.0)	96 (30.3)	58 (15.6)^a^	76 (27.0)	98 (35.4)	232 (24.9)
E-cigarettes contain tar	109 (22.8)	112 (25.9)	3 (16.7)	71 (23.7)	78 (24.8)	75 (23.7)	79 (21.3)	71 (25.2)	74 (26.7)	224 (24.1)

Values represent number (%) of participants who selected ‘Agree’ or ‘Strongly Agree’ on a 5-point Likert scale from ‘Strongly Disagree’ to ‘Strongly Agree’. Participants who responded that they did not know about e-cigarettes were excluded from these analyses.

^a^Significant difference between columns for the same variable (sex, age, smoking) at *P* < 0.001. This refers to differences between that column and all other columns for the same variable.

Over two-thirds of participants had one or two relatives who use e-cigarettes (447, 48.1% and 278, 29.9%, respectively) (Table 3). About three in four participants had up to three close friends who use e-cigarettes (one friend: 286, 30.8%; two friends: 212, 22.8%; three friends: 218, 23.4%). Participants had been widely exposed to e-cigarette advertising, including on social media (601, 64.6%); corner shops, supermarkets or petrol stations (512, 61.5%); the internet (335, 36.0%); and/or TV, streaming services or a movie at the cinema (256, 27.5%). About half of participants had been exposed to warning labels on e-cigarettes (489, 52.6%). About one in three had seen a warning label advising that e-cigarettes contain nicotine and nicotine is addictive (329, 35.4%), and one in five had seen a warning label cautioning that the long-term health risks associated with e-cigarettes are unknown (204, 21.9%).

**Table 3:** External influences by gender, age and smoking status—*n* (%)

	Women (*N* = 479)	Men (*N* = 433)	Non-binary (*N* = 18)	15–19 (*N* = 299)	20–25 (*N* = 314)	26–30 (*N* = 341)	Never smoker (*N* = 317)	Previous smoker (*N* = 371)	Current smoker (*N* = 282)	Overall (*N* = 930)
Number of family members using e-cigarettes										
None	236 (49.3)	203 (46.9)	8 (44.4)	149 (49.8)	133 (42.4)	165 (52.1)	235 (63.3)^*^	118 (41.8)	94 (33.9)	447 (48.1)
One	145 (30.3)	128 (29.6)	5 (27.8)	98 (32.8)	97 (30.9)	83 (26.2)	84 (22.6)	94 (33.3)	100 (36.1)	278 (29.9)
Two	70 (14.6)	85 (19.6)	3 (16.7)	36 (12.0)	67 (21.3)	55 (17.4)	40 (10.8)^*^	55 (19.5)	63 (22.7)	158 (17.0)
Three	21 (4.4)	10 (2.3)	0 (0.0)	10 (3.3)	9 (2.9)	12 (3.8)	8 (2.2)	8 (2.8)	15 (5.4)	31 (3.3)
Four or more	7 (1.5)	7 (1.6)	2 (11.1)	6 (2.0)	8 (2.5)	2 (0.6)	4 (1.1)	7 (2.5)	5 (1.8)	16 (1.7)
Number of close friends using e-cigarettes										
None	149 (31.1)	133 (30.7)	4 (22.2)	99 (33.1)	87 (27.7)	100 (31.5)	165 (44.5)^*^	65 (23.0)	56 (20.2)	286 (30.8)
One	109 (22.8)	102 (23.6)	1 (5.6)	52 (17.4)^*^	82 (26.1)	78 (24.6)	77 (20.8)	66 (23.4)	69 (24.9)	212 (22.8)
Two	115 (24.0)	98 (22.6)	5 (27.8)	59 (19.7)	81 (25.8)	78 (24.6)	65 (17.5)^*^	72 (25.5)	81 (29.2)	218 (23.4)
Three	39 (8.1)	37 (8.5)	1 (5.6)	19 (6.4)	27 (8.6)	31 (9.8)	22 (5.9)^*^	24 (8.5)	31 (11.2)	77 (8.3)
Four or more	67 (14.0)	63 (14.5)	7 (38.9)	70 (23.4)^*^	37 (11.8)	30 (9.5)	42 (11.3)^*^	55 (19.5)	40 (14.4)	137 (14.7)
Exposure to warning labels on e-cigarette packages^a^	238 (49.7)	242 (55.9)	9 (50.0)	148 (49.5)	167 (53.2)	174 (54.9)	160 (43.1)	140 (49.6)	189 (68.2)^*^	489 (52.6)
Types of warning										
WARNING: This product contains nicotine. Nicotine is an addictive chemical.	165 (34.4)	155 (35.8)	9 (50.0)	107 (35.8)	121 (38.5)	101 (31.9)	95 (25.6)	113 (40.1)	121 (43.7)	329 (35.4)
WARNING: The long-term health risks associated with this product are unknown.	94 (19.6)	108 (24.9)	2 (11.1)	56 (18.7)	77 (24.5)	71 (22.4)	68 (18.3)	65 (23.0)	71 (25.6)	204 (21.9)
WARNING: This product is not a smoking cessation product.	32 (6.7)	46 (10.6)	0 (0.0)	15 (5.0)	30 (9.6)	33 (10.4)	30 (8.1)	24 (8.5)	24 (8.7)	78 (8.4)
Exposure to advertising^b^										
Social media	301 (62.8)	284 (65.6)	16 (88.9)	196 (65.6)	223 (71.0)	182 (57.4)	206 (55.5)^*^	183 (64.9)	212 (76.5)	601 (64.6)
Corner store, supermarket or petrol station	296 (61.8)	262 (60.5)	14 (77.8)	187 (62.5)	180 (57.3)	205 (64.7)	198 (53.4)^*^	175 (62.1)	199 (71.8)	572 (61.5)
Internet	163 (34.0)	162 (37.4)	10 (55.6)	100 (33.4)	112 (35.7)	123 (38.8)	110 (29.6)	106 (37.6)	119 (43.0)	335 (36.0)
TV, streaming services (such as Netflix) or a movie at the cinema	127 (26.5)	123 (28.4)	6 (33.3)	73 (24.4)	92 (29.3)	91 (28.7)	75 (20.2)^*^	75 (26.6)	106 (38.3)	256 (27.5)

Participants who responded that they did not know about e-cigarettes were excluded from these analyses.

^a^Selected ‘Sometimes’, ‘Most of the time’ or ‘Always’ on a 5-point scale: ‘Never’, ‘Rarely’, ‘Sometimes’, ‘Most of the time’, ‘Always’.

^b^Selected ‘Sometimes’ or ‘Often’ on 4-point scale: ‘Never’, ‘Rarely’, ‘Sometimes’, ‘Often’.

^*^Significant difference between columns for the same variable (sex, age, smoking) at *P* < 0.001. This refers to differences between that column and all other columns for the same variable.

### Factors associated with ever use of e-cigarettes

Age and gender were not associated with ever use of e-cigarettes (Table 4). The strongest predictors of using e-cigarettes were previous or current tobacco smoking (OR 8.50 95% CI [5.15–14.04], *P* < 0.001 and 22.28 [12.19–40.71], *P* < 0.001, respectively). The likelihood of using e-cigarettes increased as the number of relatives or close friends using e-cigarettes increased (1.95 [1.49–2.56], *P* < 0.001 and 1.57 [1.32–1.88], *P* < 0.001, per each additional relative and close friend, respectively). Perceiving e-cigarettes as harmful was associated with a ~40% lower likelihood of using e-cigarettes (0.64 [0.49–0.83], *P* < 0.001).

**Table 4:** Factors associated with ever use of e-cigarettes

		Odds ratio [95% confidence interval]	*P*-value
Gender	Men	Reference	
	Women	1.37 [0.89–2.10]	0.152
	Non-binary	1.82 [0.30–10.93]	0.512
Age	16–19	Reference	
	20–25	0.98 [0.54–1.78]	0.957
	26–30	0.66 [0.35–1.22]	0.184
Education	Primary or Secondary School	Reference	
	College or Diploma	1.09 [0.61–1.97]	0.763
	University	1.29 [0.78–2.15]	0.322
Income	Low	Reference	
	Middle	1.05 [0.63–1.76]	0.843
	High	1.14 [0.65–2.00]	0.653
Smoking	Never	Reference	
	Previous	8.50 [5.15–14.04]	<0.001
	Current	22.28 [12.19–40.71]	<0.001
Number of relatives		1.95 [1.49–2.56]	<0.001
Number of friends		1.57 [1.32–1.88]	<0.001
Exposure to advertising		1.60 [0.91–2.82]	0.103
Perceived addictiveness		0.88 [0.61–1.28]	0.512
Perceived harmfulness		0.64 [0.49–0.83]	0.001

## Discussion

### Main finding of this study

In a sample of 1009 15–30-year-olds in the UK, about one in five currently used e-cigarettes at least monthly, with one in ten using them daily. Amongst vapers, e-cigarettes were similarly used by women and men and appeared to be more used by those over the age of 20 years. Current smokers were the most likely to use e-cigarettes followed by previous smokers, with low use reported by never smokers. Amongst those using e-cigarettes at least monthly, about 90% reported having used e-cigarettes containing nicotine and about one in three said they had used e-cigarettes containing cannabis. Approximately one in seven at least once monthly vapers had medical prescriptions for e-cigarettes. E-cigarettes were mainly obtained from vape shops and used at home (either their own or someone else’s), parties and the workplace. Having friends who used e-cigarettes and using them to help quit or reduce the use of tobacco cigarettes were the most common reasons for using e-cigarettes. Most participants agreed that e-cigarettes are addictive, contain chemicals and nicotine, help people quitting smoking and are bad for health. About half of participants had been exposed to warning labels on e-cigarettes and e-cigarette advertising, especially online. Previous or current tobacco smokers were 9 and 22 times more likely to use e-cigarettes than never smokers, respectively. Perceiving e-cigarettes as harmful was associated with a 40% lower likelihood of use.

### What is already known on this topic

Our estimated prevalence of using e-cigarettes at least monthly was approximately twice of that reported by the Office of National Statistics in the UK in 2021 (11% for daily or occasional vapers aged 16–34 years), when this survey was conducted.^1^ Our prevalence of vaping at least once monthly was also higher than official statistics for current (45 versus 25%) and previous cigarette smokers (29 versus 15%).^1^ Use of e-cigarettes by never smokers in our survey was low (about 2%), which is broadly comparable to the estimated prevalence from several national studies (0.7%).^23^ Taken together, these comparisons suggest that our sample might have been enriched with vapers, possibly because they were more likely to respond to this survey. It is also possible that different definitions of vaping may have contributed to the observed discrepancy in prevalence between studies. Therefore, our findings may not be generalizable to the population of 15–30-year-olds, but they may be indicative of the behaviour, attitudes and beliefs of current vapers in the UK.

### What this study adds

Our study adds to the official statistics by including under 18-year-olds, who are not included in the national survey. We found important differences between under 20-year-olds, which includes under 18-year-olds to whom it is illegal to sell e-cigarettes, and those aged 20 and over. For instance, under 20-year-olds were more likely to use disposable e-cigarettes, which are cheaper than other devices and have been identified as drivers of vaping amongst teenagers.^24^ They were also more likely to have used fruit flavours, which seem to be particularly appealing to adolescents and hence promoted by targeted marketing campaigns.^25^ Under 20-year-olds were more likely to vape at parties and because of friends’ influence as well as to have four or more friends who vaped than older age groups. This highlights the importance of peer pressure in this age group, which has been exploited by industry.^26^ Although our findings are based on under 20-year-olds, they broadly support the adoption by the UK government of evidence-based policies that address the factors that encourage vaping by under 18-year-olds, such as reducing the appeal by regulating product characteristics, forbidding advertising and raising awareness about harms in education campaigns.^27^

Dual use (i.e. concomitant use of tobacco and e-cigarettes) was very common, with ~45% of current smokers using e-cigarettes at least monthly. It is uncertain whether dual smokers started by using e-cigarettes and then transitioned to tobacco cigarettes or vice-versa. This is a relevant question for future research and policy because of ongoing concerns that e-cigarettes might be a gateway to tobacco smoking vis-à-vis the rising prevalence of vaping amongst young people in the UK.^10^ Furthermore, previous research has shown that users of e-cigarettes were two to four times more willing to try tobacco compared with those who had never used any tobacco product, which seemed partly mediated by positive expectations about smoking.^28^^,^^29^ Although recent evidence suggests that many young people who start using e-cigarettes and transition to smoking may not continue after initiation of vaping,^30^ it is important to validate these findings in other cohorts and investigate whether e-cigarette users in youth have an increased risk of becoming smokers later in life. Evidence is also lacking on whether short-term or intermittent use of e-cigarettes in youth can lead to negative health outcomes in adulthood.

Environmental concerns about vaping have been increasing recently, particularly fuelled by the rise in disposable devices.^31^ Although our cross-sectional survey cannot investigate trends over time, the finding that about three in four vapers aged 15–19 years used disposable e-cigarettes suggests they are particularly common amongst teenagers, who may keep using them if they continue using e-cigarettes through adulthood. This is supported by a recent report from the public health charity ‘Action on Smoking and Health’ in the UK, which showed that, for the first time in 2022, disposable e-cigarettes were the devices most frequently used by 11–18-year-olds in Britain (52.0% compared with 7.7% in 2021).^32^ Environmental concerns because of this recent increase in use of disposable devices, especially by teenagers, add to concerns about harmful health consequences of vaping in this population.^33^ Together they underpin measures adopted by the UK government to prevent use e-cigarettes by under 18-year-olds.^34^ These include enforcing sales and marketing restrictions, such as plain packaging, which may be especially effective at reducing the appeal to young people.^35^

The results of this study suggest that influence of relatives and friends is an important driver of using e-cigarettes. The influence of significant others, particularly friends and family, on use of e-cigarettes by young people has been documented by previous studies in other countries.^36^ Understanding how complex social networks, both in person and online (e.g. on social media), can either promote or prevent vaping amongst young people is crucial to design effective public health campaigns raising awareness on the unknown health effects of e-cigarettes and discouraging their use.^37^ Individual-based interventions that fail to account for the influence of family and friends are unlikely to succeed for young people who do not have the support network needed to stop vaping and smoking and are immersed in an environment where they are consistently exposed to e-cigarettes and/or tobacco. Therefore, interventions based in schools or targeting all the smokers and/or e-cigarette users in a household or similar social group may be more likely to be successful in the long-term.^38^

The fact that perceiving e-cigarettes as harmful was associated with a 40% lower likelihood of using them demonstrates the importance of raising awareness on the already established harms of e-cigarettes, such as vaping-related lung injury. E-cigarettes are relatively recent and, thus, their potential risks, particularly associated with prolonged used, remain poorly understood.^39^ In addition, only half of e-cigarette users and less than a third of current tobacco smokers in our study reported having seen warning labels on packages of e-cigarettes. This is in keeping with evidence from other national surveys in the UK, which showed lack of awareness about the relative risks of smoking and vaping.^23^ Taken together, these findings demonstrate the need for stronger policy on labelling along with well-designed public health campaigns to inform about the potential harms of using e-cigarettes and prevent misinformation from luring young people into vaping.

The findings of our study are relevant to inform public health policy. Despite accruing evidence on their cost-effectiveness to aid reducing or quitting tobacco smoking,^6^ controversy remains about net population benefit amidst growing concerns about long-term health implications.^40^ Contrary to other countries that, in the absence of robust evidence, have adopted a precautionary approach to e-cigarettes,^41^ the UK has widely endorsed e-cigarettes as an aid to quitting or reducing tobacco smoking. The updated NICE guidelines on tobacco smoking published in 2021 recommend giving advice on how to use e-cigarettes to smokers who demonstrate interest in using them, including that they are likely to be ‘substantially less harmful than smoking’, but evidence is lacking on ‘whether there are long-term harms from e-cigarette use’.^6^ Nonetheless, e-cigarettes were considered cost-effective and their recommendation by NICE guidelines may have influenced the Office for Health Improvement and Disparities, the public health arm of the Department of Health and Social Care, to recommend their use as a first-line therapy alongside medicinally licensed stop smoking products.^42^ Furthermore, the Crown Commercial Services added vaping product catalogues to existing public sector procurement frameworks in April 2023.^42^ This is likely to result in a significant increase in medical prescriptions of e-cigarettes, which our study suggests were rare in 2021 as only 15% of users were prescribed e-cigarettes.

The relatively lenient approach to e-cigarettes in the UK contrasts with World Health Organization recommendations to limit availability of e-cigarettes and minimize uptake because of concerns about their adverse health effects and the potential for tobacco use to be renormalized through public exposure to vaping.^43^ It is thus critical that policy and regulation are updated as evidence emerges on the long-term consequences of using e-cigarettes across the life course, particularly when used concomitantly with tobacco and by those who may be particularly vulnerable to harmful effects, such as young people.^44^

### Limitations of this study

This study has some limitations. First, it is possible that our findings are not generalizable to the general population of 15–30-year-olds even if quotas were used to obtain a roughly representative sample for demographic variables. Our study sample seems to have a higher prevalence of vaping than official statistics, which suggests our findings may be broadly generalizable to e-cigarette users but not to the general population of 15–30-year-olds. Second, many of the survey items did not differentiate between nicotine and non-nicotine e-cigarettes because of the inconsistent labelling of e-cigarettes, which meant that participants could be unsure about the contents of the e-cigarettes they used. Third, the factors associated with using e-cigarettes may not be causal as this is a cross-sectional study. Fourth, under 18-year-olds represented only about 13% of the study population and, hence, further studies are warranted in this age group.

## Conclusion

E-cigarettes seem to be used by a substantial proportion of 15–30-year-olds in the UK. Although they appear to be mainly used by current or former smokers, their regular use by under 20-year-olds is concerning. Further research is warranted to understand the drivers of their illegal use by under 18-year-olds. It is imperative that support for e-cigarettes as aids to reduce or stop smoking by public health authorities is matched by raising awareness about their uncertain impact and tight regulation on labelling, advertising and sales.

## Conflict of interest

None declared.

## Funding

This study was funded by National Health and Medical Council Grant GNT1149987. ACPG is funded by a Clinical Lectureship from the National Institute for Health and Care Research in the UK.

## Authors’ contributions

ACPG and SP designed this study. ACPG analyzed the data and drafted the manuscript. JAS reviewed the analyses. All authors reviewed and edited the manuscript.

## Ethical standards disclosure

This study was conducted according to the guidelines laid down in the Declaration of Helsinki and all procedures involving research study participants were approved by the Human Research Ethics Committee of University of New South Wales.

## Data availability

The data underlying this article will be shared on reasonable request to the corresponding author.

## Supplementary Material

vaping_supp_jph_fdad138

## Notes

**Ana-Catarina Pinho-Gomes,** Public Health Clinical Lecturer

**Joseph A. Santos,** statistician

**Alexandra Jones,** senior research fellow

**Thout S. Raj,** research fellow

**Simone Pettigrew,** professor, Program Head of Food Policy
