# Supplementary material for: E-cigarette attitudes and behaviours amongst 15-30-year-olds in the UK
Source: J Public Health (Oxf). 2023 Jul 31;45(4):e763–75. doi: 10.1093/pubmed/fdad138 (PMC10687603; doi:10.1093/pubmed/fdad138)
Supplement: vaping_supp_jph_fdad138 [file vaping_supp_jph_fdad138.docx]

**Supplementary data**

**Table S1: Characteristics of the study population**

|  | Overall (n=1,009) | Current e-cigarette user  (n=278)^1^ | Never e-cigarette user  (n=333)^1^ | Previous e-cigarette user  (n=319)^1^ | Current smoker and vaper (dual) (n=154)^2^ | Current smoker and previous/never vaper (n=123) |
| --- | --- | --- | --- | --- | --- | --- |
| Age (years) |  |  |  |  |  |  |
| 15 to 19 | 327 (32.4) | 65 (23.4) | 135 (40.5) | 99 (31.0) | 32 (20.8) | 17 (13.8) |
| 20 to 25 | 341 (33.8) | 115 (41.4) | 90 (27.0) | 109 (34.2) | 61 (39.6) | 48 (39.0) |
| 26 to 30 | 341 (33.8) | 98 (35.3) | 108 (32.4) | 111 (34.8) | 61 (39.6) | 58 (47.2) |
| Gender |  |  |  |  |  |  |
| Women | 520 (51.5) | 130 (46.8) | 175 (52.6) | 174 (54.5) | 67 (43.5) | 63 (51.2) |
| Men | 470 (46.6) | 137 (49.3) | 153 (45.9) | 143 (44.8) | 79 (51.3) | 60 (48.8) |
| Non-binary | 19 (1.9) | 11 (4.0) | 5 (1.5) | 2 (0.6) | 8 (5.2) | 0 (0) |
| Education |  |  |  |  |  |  |
| Primary or secondary school | 486 (48.2%) | 111 (39.9) | 171 (51.4) | 160 (50.2) | 60 (39.0) | 66 (53.7) |
| College or diploma | 182 (18.0%) | 55 (19.8) | 61 (18.3) | 54 (16.9) | 28 (18.2) | 22 (17.9) |
| University | 339 (33.6%) | 112 (40.3) | 101 (30.3) | 104 (32.6) | 66 (42.9) | 35 (28.5) |
| Missing | 2 (0.2%) | 0 (0) | 0 (0) | 1 (0.3) | 0 (0) | 0 (0) |
| Income |  |  |  |  |  |  |
| High | 236 (23.4) | 83 (29.9) | 61 (18.3) | 71 (22.3) | 52 (33.8) | 28 (22.8) |
| Medium | 303 (30.0) | 72 (25.9) | 105 (31.5) | 102 (32.0) | 36 (23.4) | 47 (38.2) |
| Low | 328 (32.5) | 103 (37.1) | 98 (29.4) | 108 (33.9) | 57 (37.0) | 41 (33.3) |
| Missing | 142 (14.1) | 20 (7.2) | 69 (20.7) | 38 (11.9) | 9 (5.8) | 7 (5.7) |

^1^ 79 participants responded that they had not hear about e-cigarettes and were excluded from subsequent analyses.

^2^ This includes all users of e-cigarettes irrespective of frequency (i.e., occasional and regular users).
